# Lessons learned from longitudinal biomonitoring and birth cohort studies: informing the future of reproductive health research in Canada

**DOI:** 10.1186/s12940-026-01313-8

**Published:** 2026-05-29

**Authors:** Jillian Ashley-Martin, Mandy Fisher, Cheryl Khoury, Aranee Sathiyamoorthy, Michael M. Borghese, Gillian England-Mason, Tyler Pollock, Tye E. Arbuckle, Lauren A. Wise, Joseph M. Braun, Amy Metcalfe, Jessie P. Buckley, Mark R. Palmert, Christina Ricci, Erica Phipps, Gopal Banerjee, Erin Fuller, Linda Booij, Kathyrn Hopperton, Hope Weiler, Amanda J. MacFarlane, Jesse Bertinato, Dorothea F. K. Rawn, Eric Lavigne, Amalie Timmermann, Emily Oken, Christina Wolfson, Ragnhild Eek Brandlistuen, Robin Shutt

**Affiliations:** 1 Environmental Health Science and Research Bureau, Health Canada, 219 Laurier Ave, Ottawa, ON K1A 0K9 Canada; 2 Department of Epidemiology, Boston University, Boston, MA USA; 3 Department of Epidemiology, Brown University, Providence, RI USA; 4 Cummings School of Medicine, University of Calgary, Calgary, AB Canada; 5 Department of Epidemiology, Gillings School of Global Public Health, University of North Carolina at Chapel Hill, Chapel Hill, NC USA; 6 Department of Physiology, University of Toronto, Toronto, ON Canada; 7 Department of Pediatrics, University of British Columbia, Vancouver, BC Canada; 8 Health Promotion and Chronic Disease Prevention Branch, Public Health Agency of Canada, 785 Carling Ave, Ottawa, ON K1A 0K9 Canada; 9 Department of Geography, University of Ottawa, Ottawa, ON Canada; 10 Canadian Partnership for Children’s Health and the Environment, Ottawa, ON Canada; 11 Hamilton Community Legal Clinic, Hamilton, ON Canada; 12 Hamilton Department of Public Health, , Hamilton, ON Canada; 13 Department of Psychiatry, McGill University, Montreal, QC Canada; 14 CHU Sainte-Justine Azrieli Reseach Center, Montreal, QC Canada; 15 Bureau of Nutritional Sciences, Food and Nutrition Directorate, Health Products and Food Branch, Health Canada, 251 Sir Frederick Banting Driveway, Tunney’s Pasture, Ottawa, ON K1A 0K9 Canada; 16 Food Research Division, Bureau of Chemical Safety, Food and Nutrition Directorate, Health Products and Food Branch, Health Canada, 251 Sir Frederick Banting Driveway, Tunney’s Pasture, Ottawa, ON K1A 0K9 Canada; 17 National Institute of Public Health, University of Southern Denmark, Copenhagen, Denmark; 18 Harvard Pilgrim Health Care Institute, Boston, MA USA; 19 Department of Epidemiology, Biostatistics and Occupational Health, McGill University, Montreal, QC Canada; 20 Norwegian Institute of Public Health, Oslo, Norway

**Keywords:** Biomonitoring, Longitudinal, Pregnancy, Environmental chemicals, Epidemiology, Cohort

## Abstract

Longitudinal biomonitoring studies during preconception, pregnancy and early childhood are highly valuable tools for assessing environmental chemical exposures during sensitive windows and their effects on health and development. For the past 15 years, the Maternal-Infant Research on Environmental Chemicals (MIREC) Research Platform has been Canada’s flagship study of the long-term effects of early life exposure to environmental chemicals. In light of the evolving scientific and legislative landscapes and need to address emerging research questions, MIREC Platform researchers at Health Canada consulted with scientific investigators of other cohort studies to inform the development of a future preconception or pregnancy longitudinal biomonitoring study. This effort included 1) hybrid consultation meetings on Dec 6, 2024 (Toronto, ON) and Jan 21, 2025 (Ottawa, ON) and 2) a virtual seminar series from October 2024 to June 2025 hosted by the Health Canada MIREC team. Our objective here is to share lessons learned from this consultation. We report on key lessons learned related to the themes of: 1) participant engagement, recruitment and retention, 2) validity and causal inference, and 3) study longevity. While the ultimate goal of this consultation was to inform future longitudinal biomonitoring studies in Canada, the content is largely generalizable and relevant to others planning, modifying, or evaluating observational research in reproductive and environmental epidemiology.

## Background

**Fig. 1:**
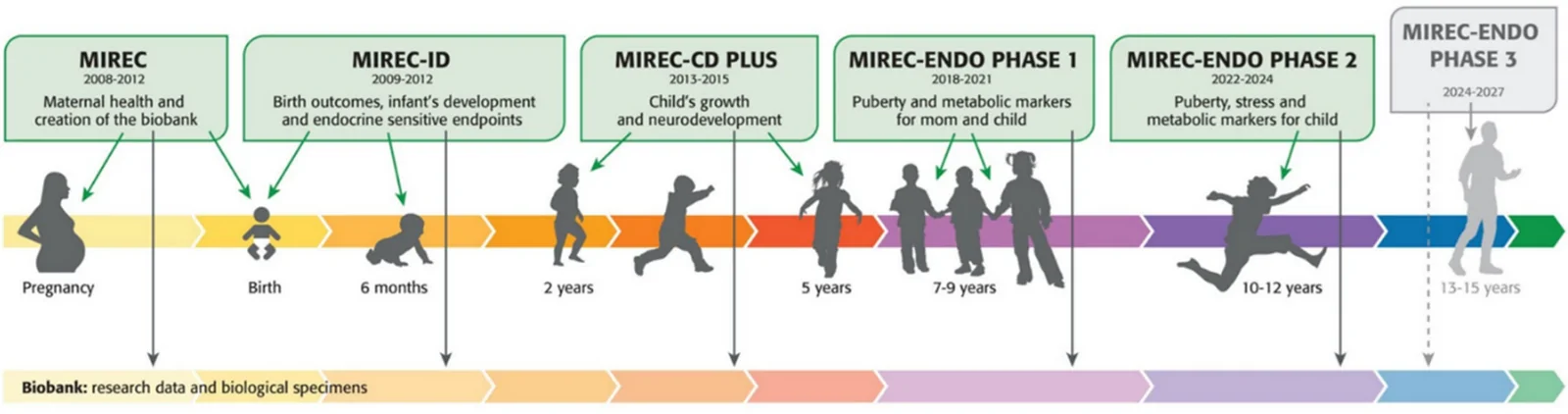
MIREC Research Platform timeline

Longitudinal biomonitoring and health outcome studies during preconception, pregnancy and early childhood are highly valuable tools for assessing environmental chemical exposures and their health effects during sensitive windows. For the past 15 years, the Maternal-Infant Research on Environmental Chemicals (MIREC) Research Platform has been Canada’s flagship study of the longitudinal effects of early life exposure to environmental chemicals. MIREC began as a pregnancy cohort that recruited approximately 2000 participants from 10 Canadian cities during their first trimester of pregnancy (2008–2011) and has since followed the original participants and their children for over 15 years [1] (Fig. 1). MIREC was conceptualized during a time of growing scientific recognition of the Developmental Origins of Disease – or Barker Hypothesis [2] – and recognition that children’s environmental health warrants a special research focus [3–5]. It was within this context that Health Canada and Environment and Climate Change Canada convened a Canadian Children’s Environmental Health Research Workshop and recommended a pan-Canadian pregnancy cohort study to address concerns over environmental chemicals [6].

MIREC researchers have since generated substantial evidence on levels of exposure to, and health effects associated with, environmental chemicals during pregnancy and childhood [7], produced the first and only biomonitoring data on multiple priority chemicals during pregnancy and early life, developed a biobank with over 300,000 biospecimens and 1,000,000 laboratory results and contributed to Canadian and international chemical risk assessments and risk management activities. The value of the MIREC Research Platform continues to grow with long-term follow-up and investigation of novel research questions regarding the interplay between environmental chemicals and onset of new reproductive milestones for mothers and youth, including menopause and puberty, respectively.

There are limits, however, to the scope of research questions that can be answered with MIREC data. Historical biospecimens cannot be used to assess contemporary exposure profiles to new and emerging chemicals of concern. Moreover, the MIREC study did not set out to specifically engage with individuals who may experience intersecting socioeconomic marginalization and environmental chemical exposure. The MIREC study participants – as with many pregnancy cohorts initiated during the same time period [8–10] – are relatively homogeneous in terms of socioeconomic status and racial and ethnic characteristics. For example, approximately 80% of participants were born in Canada and self-identified as White and the majority of participants had household income over $80,000 CAD [1, 11]. Furthermore, MIREC was not designed to assess paternal or preconception environmental chemical exposures. New research studies with novel approaches to study design are needed to address these data gaps and inform chemical risk management and assessment activities. The scientific need for these data aligns with recent amendments to the *Canadian Environmental Protection Act, 1999* (CEPA) [12].

CEPA is an important part of Canada's federal environmental legislation aimed at preventing pollution and protecting the environment and human health. This Act was amended in June 2023, and updates include the recognition that that every individual in Canada has a right to a healthy environment, as provided under CEPA, subject to reasonable limits. Amendments also required the development of an implementation framework to set out how this right can be considered in the administration of the Act. The framework identified procedural elements of the right, including access to information and participation in decision-making, and elaborated on three new principles to be upheld under CEPA: environmental justice, intergenerational equity, and non-regression (Table 1) [13, 14]. Furthermore, requirements were introduced to undertake research, studies or monitoring activities to support the protection of the right, and to conduct biomonitoring surveys, as part of the Minister of Health’s obligation to conduct research and studies relating to the health effects of substances, including specifying that these activities may relate to vulnerable populations (i.e., populations who may be disproportionately impacted) [15].

**Table 1:** New principles to be upheld in the administration of CEPA

• *Environmental Justice* seeks to advance the fair and equitable protection of all people in Canada from disproportionate environmental or health risks and to advance their equitable access to meaningful participation in decision-making under the Act
• *Intergenerational equity* emphasizes that it is important to meet the needs of the present generation without compromising the ability of future generations to meet their own needs
• *Non-regression* means to prevent reduced levels of environmental and human health protection and, where feasible, to continuously improve these levels of protection

In recognition of this legislative and scientific landscape, MIREC Platform researchers at Health Canada consulted with scientific leads of other cohort studies to inform the development of a future preconception or pregnancy longitudinal biomonitoring study in Canada. This effort included 1) hybrid consultation meetings on Dec 6, 2024 (Toronto, ON) and Jan 21, 2025 (Ottawa, ON) and 2) a virtual seminar series from October 2024 to June 2025 hosted by the Health Canada MIREC team. Participants were asked to provide details on their cohort’s study design and data collection, reflect on the successes and difficulties they encountered, and offer their advice on building a new cohort. The main themes and conclusions from these consultations were derived using an informal process. The lead author collated themes and notes from multiple team members and reviewed the recordings of all seminar series. All co-authors were present for the consultation meetings or seminar series and reviewed the content of this manuscript.

Our objective here is to share lessons learned from this consultation. We identified several primary themes: 1) participant engagement, recruitment and retention, 2) validity and causal inference and 3) study longevity. While the ultimate goal of this consultation was to inform a specific future Canadian research program, the content is largely generalizable and relevant to others planning, modifying, or evaluating observational research in reproductive and environmental epidemiology.

## Intra-study lessons learned in engagement, recruitment, retention

Recognizing that quality research hinges upon the successful engagement, recruitment and retention of participants, the importance of *cohort care* was a dominant theme in our consultations. Cohort care embodies the need to be patient, polite and precise and the ‘*We are here for you*’ attitude. As noted by Dr. Pál Weihe of the Faroe Islands cohorts, ‘*All participants are treated like precious customers – not patients*.’ Building upon this overarching theme, we identified five key principles to facilitate successful cohort care (Table 2). While there is overlap among these principles, we provide our interpretation and specific examples below.

**Table 2:** Principles to facilitate successful cohort care

• Branding
• Listening
• Adapting
• Reciprocating
• Connecting

### Branding

Research may benefit from leveraging marketing and social media strategies to make the research relevant to and engaging for prospective participants.

The Faroe Islands studies provide a compelling example. A total of 3000 mother–child pairs were recruited into six prospective birth cohorts in the Faroe Islands. The earliest cohort recruited 1022 pregnant participants in 1986–1987. Over 700 of these children returned for a follow up visit in 2014–2015 representing a nearly 70% retention over 28 years [16]. The high participation and retention in these studies can be partially attributed to Pál Weihe. He is well respected in the community, and has been the Medical Director of the hospital system and Chair of the Faroese Board of Health. He has personally reached out to community members to invite them to participate in the research. His role as a well-known and trusted ‘public health celebrity’ enhances prospective participant’s trust and interest in participating. (As an example of his face recognition, he has been featured on milk cartons encouraging people to drink milk). Although this example may be unique to the Faroe Island context, and difficult to replicate in study populations that are more geographically and demographically heterogenous, it does demonstrate the power of building a research ‘brand’ that has a local centre of gravity and a familiar and trusted lead.

More contemporary cohorts are effectively using social media to generate interest, recognition and cohesion [17–19]. The Calgary (Alberta, Canada) based P3 Cohort study [17], for example, posts recruitment advertisements on Instagram and Facebook as well as engaging updates for current and prospective participants. These include pictures of research leads, new cohort babies, and posts celebrating events such as International Women’s Day. This type of engagement creates a sense of virtual community for current and prospective participants and may enhance long-term retention particularly if the study communication strategies and incentives adapt to the relevant life stage of the cohort.

While branding can build a sense of community, the brand must evolve with the cohort. As pregnancy cohorts ‘grow up’, the messaging and engagement strategies need to shift from a focus on early life development to middle childhood, adolescence and beyond [20]. In addition to the evolving focus on the offspring, continued engagement with mothers requires adapting research, messaging and engagement to their health issues in midlife. The Pregnancy Study Online (PRESTO), a US web-based preconception study [21], for example, just launched a 10-year follow-up of couples enrolled in the study with a focus on medical conditions common in mid-life (e.g., hypertension, diabetes, autoimmune diseases), as well as menopausal symptoms and weight change. Keeping the study focus relevant to the changing ages and life stages of participants is critical for ongoing engagement and perceived relevance.

Branding has also been used to address attrition. Project Viva study (Boston, MA, USA) researchers noted higher attrition in male adolescent and young adult participants than females [22]. In response, the Project Viva team updated their communication materials, including the website, to include pictures of boys and young men – not just pregnant women. In addition to enhancing engagement, branding may be an effective tool for making research relevant to participants who have traditionally been underrepresented in environmental health research. Two important subgroups that were discussed in our consultations were fathers and marginalized communities.

#### Reaching fathers

Despite the importance of paternal environmental chemical body burden on couple-based outcomes such as fecundity [23, 24], researchers have been challenged to successfully engage fathers in environmental and reproductive health research [25–27]. The P3 pregnancy cohort study (recruitment 2021–2025) recruited 43% of fathers/partners that were invited by their partners [28]. In the PRESTO study, 57% of female participants invited their male partners to complete a baseline questionnaire; of those, 50% agreed to participate. Partner involvement differed by socioeconomic status and race and ethnicity; with married, more educated, White women more likely to invite their partners [21]. European studies, such as the Dutch Generation R (GenR) [29] and the Norwegian Mother, Father and Child Cohort (MoBa) [30], have demonstrated success in recruiting fathers – and even collecting biological specimens – but have measured environmental chemicals in those specimens to a limited extent, in part because environmental chemicals were not the main focus of the research platforms. The lack of paternal biospecimen data is a noted gap in the field of reproductive health research [31].

One of the determinants of this gap is the persistent view that fertility is a woman’s health issue, difficult for men to engage in or discuss and, therefore, not wholly relevant to fathers [25]. Put simply, the traditional view is that men are not expected to be engaged and therefore do not engage [26, 32]. Branding the research in a way that enhances relevance to a prospective father may motivate paternal engagement and mitigate this dogma. PRESTO researchers, for example, piqued the curiosity and engagement of male partners by conducting a sub-study on semen quality and providing at home sperm tests [33]. Framing the research in terms of health of the couple or family rather than through the lens of women’s health is another successful strategy. The Longitudinal Investigation of Fertility and the Environment (LIFE) study (2005–2009), one of the few preconception studies with biomonitoring data in both partners, successfully recruited and retained both male and female partners. Using a population based sampling frame in two US states (Michigan and Texas), they recruited 501 couples and reported that male partners’ degree of participation was comparable to female partners for multiple study components including completion of baseline interviews (100% males, 100% females), daily journals (84% males, 87% females) blood collection (99.4% males, 99.8% females), and urine collection (100% males, 100% females) [34]. The P3 and PRESTO research teams similarly found that participants whose partner also participated were less likely to drop out of the study [35, 36]. Project Viva researchers attempted to contact fathers in a follow-up effort but encountered challenges; some mothers did not want the study team to reach out to fathers and some youth had no ongoing relationship.

Future research may benefit from intentionally framing reproductive health as health of the family or couple rather than just the mother and engaging with fathers directly at the outset of the study design to determine how to maximize participation.

#### Reaching marginalized communities

As previously noted, many North American pregnancy cohorts have recruited relatively homogenous study populations. As a result, study findings may not be generalizable to individuals experiencing intersecting health risks and environmental chemical exposures. Furthermore, homogenous study populations preclude disaggregated data analysis designed to assess exposures and health risks in population subgroups. Barriers to involvement may be related to lack of trust, social inequities, cultural relevance or participant burden. Designing biomonitoring research that is relevant and accessible to communities experiencing marginaliz*a*tion requires concerted efforts throughout the life course of the study to co-create strategies to mitigate these barriers. Strategies may include minimizing participant burden, addressing social inequities, building trust and being culturally relevant and safe [37–41].

**Fig. 2:**
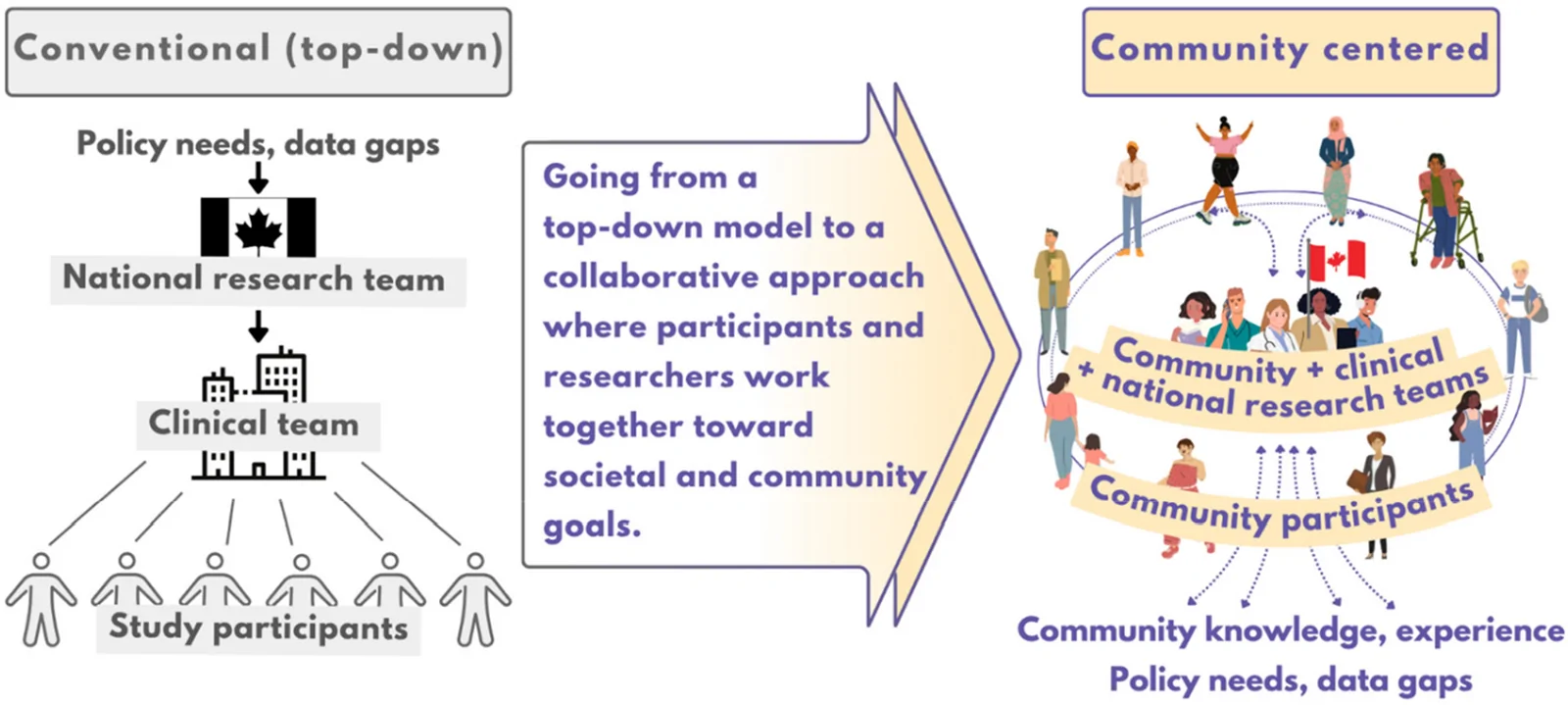
Reimagining environmental health research

Health Canada researchers have initiated the *Reimagining Research* feasibility study to further explore how to make biomonitoring research more inclusive, relevant and without harm to communities typically underrepresented in biomonitoring research (i.e. low-income, racialized, or newcomer (immigrant) [42]). Researchers found that there was notable support from community service providers and community members for a shift from a top-down traditional model of study design towards a community-centred approach that would involve ongoing exchange, collaboration, and reciprocity among researchers and the community (Fig. 2). Consistent with the principles of community-based participatory research [43, 44], the success of this model of research hinges upon trust-based partnerships between the community and the research team to develop and co-implement research. In the case of community-centred research, branding may include building on relationships between community and established community service providers, or anchoring the study with a trusted partner/provider in the lead role.

### Listening

Participant input throughout study conceptualization, design and implementation can enhance the meaning and relevance of research to participants. Participant input can also be a valuable means for identifying and responding to barriers to participation.

#### Participant advisory boards

Several cohort studies have initiated focus groups, participant advisory boards, and closed Facebook groups with both mothers and youth [17–19, 30]. By receiving input on study-related motivators and barriers to participation, study teams have had the opportunity to respond to participant priorities and concerns. For example, the P3 team launched a sibling study following interest in understanding the impact of siblings on child development. Similarly, investigators of the Alberta Pregnancy Outcomes and Nutrition (APRoN) study, another Canadian pregnancy cohort study [9], made resilience a focus of their 15-year follow up based on advisory board feedback and team discussions. MIREC has also initiated a youth advisory council to receive input on planning for a follow-up study focused on adolescent health, including timing of puberty.

These efforts also allow participants the well-appreciated opportunity to connect with each other and be part of a community within the research study. P3 researchers initiated a Facebook group just for partners, as well as for parents of preterm birth infants and for participants based on the year they delivered (i.e. 2022 babies, 2023 babies etc.). Participants used these groups to find new friends who also are new mothers and to organize local meetups amongst themselves. These groups were launched to respond to feedback that participants were feeling lonely and isolated in their new roles as parents. These Facebook groups gave the P3 team the opportunity to ask simple, specific questions such as how often the participants want to hear from the study. Going forward, P3 researchers will investigate whether participation in these groups helps promote more of a connection with the study and ultimately enhance retention. These initiatives – particularly those that involve interaction among participants – represent a shift in ethical and scientific integrity perspectives from concern about preserving participant privacy and confidentiality towards enhancing engagement and the value of fostering research as a community in and of itself. It is worth noting, however, that groups formed around ‘parenting’ or ‘birth cohort’ status may pose psychological hardships for some participants in preconception cohort studies where not all couples conceive and/or some couples experience pregnancy loss during the study period.

As another example, the community advisory board for the Health Outcomes and Measures of the Environment (HOME) Study, a Cincinnati, US based cohort study [45], provided guidance on recruitment strategies; specifically, they suggested a ‘run-in’ period so women could discuss the study with family and friends before committing to participate. This strategy allowed potential participants the time to think about participating before investing their – and the study staff’s—time in completing consents and questionnaires. Once under way, the HOME Study team demonstrated reciprocity by providing baseball game tickets and an event at the Cincinnati Zoo to participants. This event also met the goal of fostering a sense of community while also allowing participants to either self-identify or remain anonymous. This type of approach is likely to be most effective in studies such as HOME or P3 where participants are based in one city.

#### Qualitative research

During the study offers another valuable means of gaining participant feedback. PRESTO study researchers conducted interviews and focus groups with male and female participants to understand factors related to participating in fertility research. In addition to identifying barriers to participation, the researchers learned that male partners may be motivated to participate by the desire to support their partner and opportunity to gain knowledge about fertility issues [25].

Project Viva researchers conducted focus groups with mothers and adolescent participants to learn more about facilitators and barriers to ongoing participation. Both reported that supporting science was a major facilitator to remaining in the study and time constraints were an important barrier. Both groups desired more information about the scientific findings that had resulted from the study. Incentives were especially important to motivate youth participants, although the form of the incentive changed over time. For example, while books and gift cards to toy stores were appropriate in early childhood, teens were more interested in cash. At all time points, participants appreciated tangible reports of their involvement such as their bone scan images and results.

### Adapting

Flexible study designs that can adapt to a participant’s capacities, interests and ages may promote engagement, recruitment, and retention by optimizing the balance between data collection and participant burden. The need for, and benefit of, being flexible was exemplified during the COVID-19 pandemic. MIREC researchers, for example, pivoted from in-person recruitment and clinic visits in the MIREC Pubertal Timing, Endocrine and Metabolic Function (MIREC-ENDO) follow-up study to a remote questionnaire for puberty follow-up. Quickly pivoting to this global phenomenon facilitated recruitment of 273 children who otherwise may not have been included in the study [11].

#### Remote recruitment and data collection

Are feasible strategies to minimize burden while enabling participation. Remote recruitment through social media and targeted website advertising has been successfully performed in both preconception and pregnancy studies [21, 28] as determined by recruitment yield, cost-effectiveness, and participant burden. In PRESTO, Facebook was the highest yield in terms of cost and resulting recruitment [21] whereas in P3, a newer cohort, Instagram and Facebook were both cost-effective platforms for recruitment [28].

In the PRESTO study, 89% of screened individuals met the eligibility criteria (i.e. planning pregnancy, age requirements). This yield suggests that internet-based recruitment is an effective means of targeting pregnancy planners [21]. Furthermore, although internet-based studies tend to yield sociodemographically homogenous populations, the P3 study identified that socioeconomic status did not differ by recruitment from social media vs traditional methods (posters and postcards) [28].

Although not experienced by all web-based studies, some researchers have found that internet-based recruitment may come with issues related to fraud [46]. Following initial recruitment efforts, 78% of consents received by the P3 team were fraudulent. Subsequent to this experience, the P3 team implemented fraud-mitigation measures such as CAPTCHAs, IT monitoring, cross-checking data, IP addresses, verification via personal health numbers and linkage to a province-wide electronic medical record system [28].

Remote biological specimen collection also offers the possibility of greatly reducing participant burden. Both the US Environmental Influences on Child Health Outcomes (ECHO) consortium [47, 48] and the PRESTO study [31] are implementing self-administered data and biospecimen collection including blood samples for measurement of environmental chemicals. PRESTO researchers, for example, demonstrated that mail-based biospecimen collection of both blood and urine is feasible and of comparable cost to in-clinic collection [31]. Similarly, the APrON study successfully piloted remote biological sample collection during a COVID-19 sub-study and has since moved towards mail-based biospecimen collection as part of their larger and continued follow-up. Challenges of remote specimen include variable time that samples are in transit and temperature control as well as constraints on sample processing and biobanking. Nevertheless, remote specimen collection represents a feasible and promising method to enhance the number of participants who are able to provide biospecimens. In contrast to remote collection where participants mail in their biospecimens, home-based biospecimen collection is a strategy for minimizing participant burden that requires study staff to collect biospecimens in participants’ homes. While it offers the advantage of putting the travel burden on the research team rather than the participants, it requires sufficient number of staff in geographic proximity to participants and mutual feelings of safety and comfort from both participants and staff.

#### Integrating sub-studies

Offering differing levels of involvement is another means of enhancing participation among individuals with constraints in their ability to participate. The P3 study team has conducted several optional sub-studies focused on siblings, high risk pregnancies, brain health, and depression. They also initiated a novel sub-study design to enhance engagement among individuals with low-income, which was designed to evaluate the impact of a small, unrestricted cash transfer ($100 per month via electronic gift cards) on health outcomes collected via routine administrative health data for low-income pregnant people. Considering that the success of these sub-studies – each of which has its own eligibility criteria – is dependent upon ongoing recruitment into the full cohort, cohort care for the full cohort remains a priority.

Randomized controlled trials (RCT) are another effective, powerful research design that can be nested into the full cohorts of sufficiently large size and are less subject to the selection bias inherent in an optional sub-study. The HOME Study was initiated as a RCT to assess the impact of environmental lead and injury hazard controls on children’s blood lead levels and risk of injuries [45, 49]. In contrast, PRESTO researchers used a RCT after the study was initiated to assess how providing home pregnancy tests to participants impacted cohort retention and pregnancy detection [50] and to assess the extent to which randomization to a premium fertility-tracking app (FertilityFriend.com) increased fecundability [51].

The ECHO Consortium plans to leverage a case-cohort design to maximize study power without needing to analyze biospecimens from every participant [52]. In this design, all pregnant participants provide biospecimens during pregnancy and all child participants are followed for development of child health outcomes. Once children are old enough to meet case definitions for specified child health outcomes, a random sample of all children will be selected and augmented with additional participants who met the case definition(s) during follow-up. Biospecimens from this case-cohort sample will be analyzed for environmental exposures of interest. Associations of environmental exposures with the specified child health outcomes, as well as other outcomes, can be estimated by applying statistical weighting to account for oversampling of cases. This cost-saving approach is particularly well-suited to studying rare outcomes within a large cohort [52].

#### Leveraging existing data

Leveraging and linking to data collected for other purposes (e.g. administrative databases, birth registries) greatly increases the breadth of questions that researchers can ask and the availability of existing data at a low cost [53, 54]. Linkages with health administrative data can leverage the identification of rare diseases, such as childhood cancers, allows evaluation of healthcare burden over time, and enables examination of long-term health outcomes. Data linkage may also limit loss to follow-up, and support cohort care by reducing unnecessary follow-up. The value of data linkage is also seen where large but ‘shallow’ datasets such as electronic medical records (e.g., physician visits, hospitalizations, prescriptions) can enrich targeted smaller datasets of, for example, participants with extensive, resource intensive data. Multiple studies have used data linkage approaches and they are most feasible when consent for linkage is sought at the outset of the study and most valuable for health outcome data that are routinely collected in administrative data. Linkage with environmental data is also a possibility and typically does not require upfront consent; the Canadian Urban Environmental Health Research Consortium (CANUE) (canue.ca) is a possible resource for this type of linkage.

#### A priori* and *post hoc* oversampling*

Oversampling has been a successful strategy for ensuring that certain populations are represented in a study. The HOME study successfully oversampled Black participants at the study baseline in their investigations of childhood lead exposure [49]. Oversampling in the PRESTO increased the percent of self-identified racialized participants in the mail based collection protocol from 15 to 33% [55].

### Reciprocating

In addition to compensating participants for their time and involvement, sharing what the study has discovered is a critical cohort care strategy and helps participants understand the significance of their involvement. Researchers and ethics boards increasingly recognize the value of and ethical responsibility to provide results back to participants. At the time of the original MIREC study, the primary research ethics board did not permit return of individual level results for which there were no health based guidelines. Their rationale was that the ethical principle of non-maleficence outweighed the principle of autonomy and beneficence. There was concern that providing individual results without being able to contextualize the potential risks would create more anxiety than benefit [56]. Since that time, reporting results back to participants has become more clearly established as an ethical responsibility and a key component of biomonitoring research [57, 58]. The PRESTO study has used the Digital Exposure Report Back Interface (DERBI) developed by the Silent Spring Institute [59] to share individual level biomonitoring results with participants. Results are contextualized within the larger population and national levels and supplemented with information on how to reduce exposure levels. PRESTO researchers found that giving back to participants fosters trust and increases participation. Other studies, such as Project Viva, HOME, and APrON, have found value in providing results of images such as bone scans or brain anatomical images. These experiences are aligned with conclusions from a 2023 workshop organized by the National Institutes of Health confirmed the value of returning individual level research results to pregnant or pediatric participants particularly when delivered in a flexible platform and partnered with information on strategies for reducing exposures and associated risk [60].

Providing aggregate results in the form of social media posts, website infographics, or lay summaries of publications is a more common and feasible approach for sharing information. Lay abstracts of study publications are a common strategy used by multiple studies; however, it is a challenge to create a product that meaningfully represents the science and is relatable to participants. For example, a Project Viva youth participant noted ‘there’s a bunch of publishing I didn’t understand – none of them.’

### Connecting

Across all these principles of branding, adapting, listening, and reciprocating is the need for trust-based connection between participants and study staff. In addition to the aforementioned efforts to foster connection between researchers and participants and among participants, the value of engaged, long-term staff cannot be overstated. As noted by one researcher, people will not remember what they did in a study but they will remember whether they had a positive interaction with the research staff. Retention, and sufficient training, of staff is just as important as retention of participants.

Dedicated effort and time are needed to maintain contact with participants as the study progresses to minimize attrition and resulting potential selection bias. Researchers need to make best efforts to maintain up to date contact information by asking for alternate contact information, conducting internet searches or using tracking experts. Consistent with the principle of meeting people where they are at, the Project Viva team has conducted travel visits to cities around the US to allow participants who used to live locally the chance to continue to participate. Additionally, the HOME Study pays for participants to fly back to Cincinnati for study visits. Study staff need to be dedicated detectives to maintain up to date contact information. In the case where effective branding includes creating a relatable connection with the principal investigators of the study (as with the Faroese cohorts), branding and connection may be co-implemented. In the case where this is not possible, or in cases where experienced study staff cannot be retained long term, creating a study culture that includes a focus on cohort care and respectful, open, honest communication may contribute to fostering connection with the study, even if the individuals involved change.

In summary, though presented separately, these five principles are closely interconnected and all necessary to facilitate a study culture where participants feel a sense of affinity to the research. The logistical efforts required to implement and track these principles ideally translate into a sense of collective belonging to the study as a community in and of itself. Though less tangible, this affinity to the research and creation of research as a community is a key research ingredient that is sustainable and inclusive of typically underrepresented individuals. Cohort care, therefore extends from being patient, polite and precise to building reciprocal trust-based relationships. This is the type of work that reminds researchers that – as Irving Selikoff noted—the people behind our data tables are real though their tears have been wiped away [61]. The value of restoring and maintaining trust in public health cannot be overstated; fulsome implementation of cohort care, therefore, has implications beyond the objectives of a specific research project.

## Inter-study lessons learned to enhance validity and promote causal inference

### Validity

A recurring theme in our meetings was the need for high quality data with minimal bias and measurement error. Although this need is present in all epidemiological research, it is particularly pronounced in reproductive epidemiology given the finite window of key endpoints which often manifest prior to recognition [62]. Investigating environmental determinants of fecundity and fetal loss, for example, requires careful attention to timing of participant recruitment and accurate assessment of environmental chemical exposures and outcomes. Biospecimen collection from both parents prior to conception will help ensure temporality between exposures and outcomes; however, the largely unobservable nature of conception makes preconception recruitment and biospecimen collection challenging undertakings. Researchers at our meeting discussed the trade-offs between selection bias and measurement error in preconception study designs and recruitment settings (e.g. online [21], fertility clinic [63], interpregnancy [64], population based [34]). Researchers also discussed the challenges in measuring chemicals, particularly non-persistent chemicals, during the critical window of exposure and noted the value of serial biospecimen collection and pooling [65]. In addition to robust assessment of exposures and outcomes, our consultation highlighted the importance of designing studies to robustly capture factors (e.g., nutrition and physical activity) that may mediate or modify associations between environmental chemicals and health outcomes.

Notwithstanding these challenges, there was consensus that investigating environmental determinants of reproductive outcomes are research priorities. Despite the recognized multi-faceted burden of fetal loss, for example, [66] evidence on risk factors other than maternal history and advanced age is scarce [67]. Epidemiological evidence on known developmental toxicants such as certain solvent metabolites is also scarce [68]. As one researcher noted ‘mortality is our most critical outcome’ in reproductive epidemiology. In the absence of epidemiological data on fetal loss, risk assessors are required to rely on experimental evidence of chemical-induced mortality in animal models. The onus is on the research community, therefore, to fill these gaps while employing best practices to maximize internal validity.

### Causal inference

Biomonitoring research, specifically the measurement of environmental chemicals in human biospecimens, largely has either descriptive or etiological goals. We note that the rise of machine learning has led to increasing reliance on prediction models; however, our interest is primarily in etiological research that aims to identify potentially causal relationships rather than to predict outcomes. The collective goal of etiological research is to address the question of, in the words of Bradford Hill, ‘in what circumstance can we proceed from observed association to verdict of causation?’ [69]. Individual research studies are responsible for addressing principles relevant to intra-study design (e.g. temporality, dose–response); but cannot fulfill the principle of consistency: ‘Has the association been repeatedly observed by different persons in different places, circumstances and time?’ This question justifies the presence of multiple cohorts investigating similar questions with study designs customized to local conditions.

Consistency can be obtained by replication of findings by independent researchers conducting parallel research. Continued efforts to make data FAIR (findable, accessible, inter-operable, reusable) [70] and conduct pooled or federated analyses are valuable for further assessing consistency across studies and increasing statistical power to analyze mediation, moderation, and rare outcomes. Due to the known ethical and legal challenges to data sharing and pooling, a priori planning to harmonize data may mitigate some of these challenges. The MIREC study benefited from study planning on consultation with leads of the HOME study and researchers have been able to conduct pooled analyses of MIREC-HOME data [71, 72]. The ECHO Consortium is currently collecting similar data across all cohort sites in its second phase of data collection. As cohorts age and change, there are also opportunities to harmonize with past cohorts. For example, the Canadian Longitudinal Study on Aging (CLSA) [73] is collaborating with the Canadian Partnership for Tomorrow project, a research platform of multiple regional cohorts [74], to harmonize data collection as these regional cohorts age. It is, however, worth noting that although harmonization has several benefits, there is also great value in building cohorts and conducting studies with different designs as it will prevent us from reproducing biases across studies and it provides evidence from multiple contexts.

## Study longevity

Experts in our consultation noted the need for cohort studies to be innovative, impactful and sustainable. The most challenging of these pillars is sustainability. Planning a cohort study is akin to epidemiological forest planning. It is a long-term investment that needs continuous nurturing and development to provide the raw materials that may be requested in the future. Initial decisions regarding leadership, strategic planning and participant burden are all of critical importance to the successful sustained implementation of a cohort.

### Leadership

Long-term success will be enhanced by intentional development of multi-disciplinary leadership teams, early consultation with experts outside the study, and dedication to training the next generation of scientists. The HOME, MIREC, APrON, CLSA and GenR [29] studies demonstrate the value of either a multi-PI framework or having PIs or theme leads dedicated to specific outcomes which allows them to have non-competitive and non-overlapping areas of focus. Project Viva demonstrated the value of developing an advisory board of researchers early in the project to guide recruitment and retention strategies. This type of advisory board can offer a mentor–mentee relationship between researchers with experience from ‘older’ cohorts and those that are just starting. Moreover, as birth cohort studies age, there is a need to onboard new leadership and expertise into the research team to support the development of new primary objectives and to reflect the changing life stages of the cohort. For example, as the cohort grows over time, the expertise of obstetricians will need to be complemented with expertise in areas such as pediatric endocrinology and women’s health. Researchers are encouraged to identify these future expertise gaps and corresponding network of co-investigators. Mentoring junior scientists meets the shared goals of carrying forward the tradition of academic mentorship and preparing the future leaders of these cohorts. Researchers in our consultation noted the benefits of working with and learning from talented junior scientists and trainees.

### Strategic planning

Intentional and early development of the cohort-specific vision, goals, and policies is a key ingredient for study longevity. In addition to identifying scientific priorities, this planning is necessary to establish organizational structure and management frameworks. The US ECHO cohort, for example, has developed models for implementing science in the context of a multi-site research consortia [75]. A priori planning is also necessary to ensure efficient, productive inter-disciplinary collaboration and co-authorship. To this end, the MIREC Research Platform knowledge translation policy provides an exemplar framework for promoting inclusive collaboration.

MIREC, CLSA and MOBA were all established as research platforms thus allowing external researchers to investigate questions outside the initial scope of the study but requiring up front investments and sustained infrastructural, personnel and financial supports for managing these requests. An important component of the MIREC Research Platform, for example, is the MIREC Biobank which includes previously collected study data as well as biospecimens from the initial pregnancy cohort and subsequent follow-up studies. A priori establishment of this biobank has facilitated efficient, cost-effective use of data and biospecimens to investigate exploratory research questions, adapt to emerging research needs and provide valuable insights without additional costs.

Biobank leadership to guide the development of biobank objectives and criteria is critical for managing biospecimen access and balancing proximal data generation with preservation for future, unforeseen needs. Maintaining a high standard of research for biospecimen access helps achieve this balance. Table 3 provides an example of how the MIREC Biobank has defined scope and access criteria.

**Table 3:** MIREC biobank scope and access criteria

The objectives of the biobank is to provide a basis for future research on:
1. Maternal and child exposure to priority environmental chemicals
2. Fetal growth, pregnancy and the health of mothers and their infants/children
3. Health risks, if any, that are associated with various measures of chemical exposures
4. Potential mechanisms of toxicity and markers of susceptibility for adverse pregnancy and child outcomes
Criteria for granting access:
1. Feasibility
2. Scientific value
3. Minimal risk

Policies specific to which biospecimens can be released to researchers also guide the balance between current and future needs. The MIREC Biobank policy requires researchers to analyze all available biospecimens in the aliquot from the requested time point. This policy minimizes bias due to sub-sampling, ensures uniformity in the type of data available for MIREC participants and is feasible for a study population the size of MIREC (~ 2000) but would be overly restrictive and cost-prohibitive for in larger studies. The ECHO Consortium, as previously described, and other studies [76–78] are implementing nested case-cohort or case–control study designs to balance the need for unbiased sub-sampling with the cost of analysis [52, 79].

### Study design and participant burden

As previously discussed, our consultation identified principles for promoting and maintaining cohort care. Mindful attention to these principles during initial study planning will help maintain sustainable levels of participant burden while ensuring that primary study goals and objectives are met. Researchers are encouraged to identify key data points that require in-person collection versus those low-touch data points that can be obtained through leveraged, passively or remotely collected data. For example, identifying outcomes that are stable over time and measuring them less frequently reduces the need for repeated in-person measurements. Identifying data points that require in-depth, in-person assessment remains essential for maximizing data quality. Our consultation identified that future studies will benefit from a combination of low-touch approaches that maximize sample size and leverage existing data collection approaches with in-depth approaches that prioritize targeted quality data collection and participant engagement.

Being mindful of participant burden is essential not only for the success of the ongoing data collection but also for long-term engagement and affinity to the research. Even if a participant does not consent to a specific component of a follow-up study, maintaining contact and rapport with that participant is essential potential future follow-up studies. Our consultation discussed the power of continuing to leverage and learn from cohorts as they age and evolve into a multi-generational platform. Multigenerational cohorts are scarce yet suggestive of the need for further inquiry into the impacts of prenatal environmental chemical exposures on first generation fecundity and second generation health [80].

## Conclusions

Our consultation identified three overarching lessons learned for future longitudinal biomonitoring research:

1. promote participant engagement, recruitment and retention through cohort care;
2. implement data collection procedures that enhance validity and causal inference; and
3. promote study longevity through practices in leadership, strategic planning, and study design.

Integrating these lessons into the future longitudinal biomonitoring research in Canada will help address identified priority research gaps. These gaps are the scarcity of data on priority environmental chemical exposures and health effects during preconception, in prospective fathers, and among communities who may experience marginalization.

**Fig. 3:**
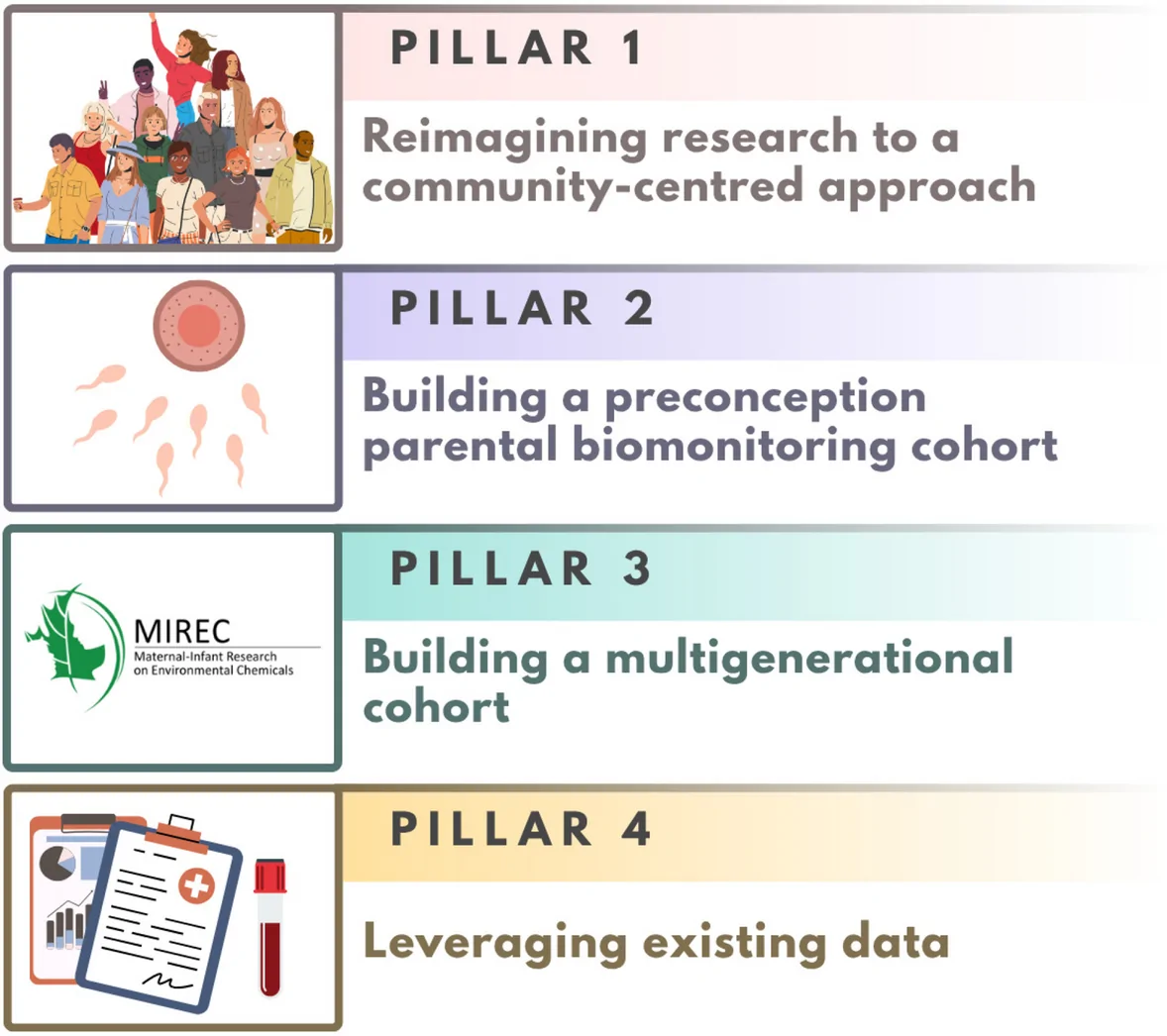
Multi-pillared approach to inform for the future of longitudinal biomonitoring research in Canada

This feedback from our consultation can inform the future of longitudinal biomonitoring and reproductive epidemiological research in Canada. We propose a multi-pillared approach that combines both low-touch and in-depth studies that are rigorously designed and sufficiently powered for the relevant research goal (Fig. 3). Our overarching goals for this research platform are to generate biomonitoring and etiological health outcome data during susceptible life stages and in disproportionately exposed populations that will inform Canadian risk assessment and risk management activities. We provide a brief high-level introduction to these pillars below. When planning priority measures for each pillar, we will aim to optimize the balance between quality science and burden to participants as well as staff. By combining primary data collection with existing secondary data, we will aim to address trade-offs between internal validity and generalizability.

### Pillar #1

Reimagining reproductive environmental health research to a community-centred approach.

#### Research goal

To develop a multi-site longitudinal investigation of levels of environmental chemical exposures and health effects in pregnant people and their partners who may experience marginalization (e.g. newcomer status, low-income, racialized) (Fig. 2).

#### Rationale

Existing literature and our consultation confirms that individuals experiencing marginalization have been typically underrepresented in research thereby limiting data and subsequent contributions to regulatory frameworks on populations who may have elevated exposure or increased health risks.

#### Study design

Multi-site, community-based research upholding principles of trust-based connections, reciprocity and inclusivity.

### Pillar #2

Building a preconception parental biomonitoring cohort.

#### Research goal

To develop a web-based longitudinal investigation of environmental chemical exposures and health effects in pregnancy planners using remote biospecimen collection.

#### Rationale

Preconception biomonitoring data, particularly in prospective fathers, is limited thereby precluding robust investigation of critical reproductive health endpoints (fetal death). Our consultation identified that a web-based approach is a feasible approach for recruiting pregnancy planners, that remote biospecimen is worthy of further exploration and that fathers are underrepresented in existing research.

#### Study design

National-level, longitudinal web-based preconception cohort that upholds principle of reciprocity and leverages existing data, including administrative databases and birth registries and using novel methods for statistical analysis.

### Pillar #3

Building a multigenerational cohort.

#### Research goal

To continue follow-up of MIREC youth participants into the reproductive years and facilitate investigation of multigenerational effects of environmental chemicals.

#### Rationale

Our consultation identified the value and scarcity of multigenerational studies with human biomonitoring data. Continued follow-up of the MIREC cohort is cost-effective and leverages the decades of planning and investment into the cohort to date.

#### Study design

Continued engagement with and follow-up studies in MIREC Research Platform mothers and youth with the long-term goal of investigating links between prenatal environmental chemical exposures in the original MIREC participants and grandchild health.

### Pillar #4

Leveraging existing data.

#### Research goal

This pillar will be a cross-cutting theme of the future of longitudinal biomonitoring and aim to identify administrative data that can be linked with cohort data to maximize data collection and research questions.

#### Rationale

Linkage will facilitate investigation of additional research questions without the need for primary data collection, integration into the national surveillance system environment and will complement national surveillance efforts. For example, linking in-person data collection with hospital records data will facilitate investigation of multiple additional endpoints.

Each of these pillars would contribute data and biospecimens that address key gaps in the field of reproductive and environmental epidemiology.

## Data Availability

No datasets were generated or analysed during the current study.

## References

[1] Arbuckle TE, Fraser WD, Fisher M, Davis K, Liang CL, Lupien N, et al. Cohort profile: the maternal-infant research on environmental chemicals research platform. Paediatr Perinat Epidemiol. 2013;27(4):415–25. 10.1111/ppe.12061 23772943

[2] Barker DJP. The origins of the developmental origins theory. J Intern Med. 2007;261:412–7. 10.1111/j.1365-2796.2007.01809.x 17444880

[3] Hudak MLPC, Annett RD, Hale DE, McGovern PM, McLaughlin TJ, Dole N, et al. The National Children’s Study: An Introduction and Historical Overview. Pediatrics. 2016;137:S213–8. 10.1542/peds.2015-4410B PMC4878112 27251867

[4] Weiss BLP. The developing brain and the environment: An introduction. Environ Health Perspect. 2000;108(Suppl 3):373–4. 10.1289/ehp.00108s3373 PMC1637828 10852830

[5] Kimmel CA, Collman GW, Fields N, Eskenazi B. Lessons learned for the National Children’s Study from the National Institute of Environmental Health Sciences/U.S. Environmental Protection Agency Centers for Children’s Environmental Health and Disease Prevention Research. Environ Health Perspect. 2005;113:1414–8. 10.1289/ehp.7669 PMC1281290 16203257

[6] Government of Canada. ARCHIVED - National Strategic Framework on Children's Environmental Health. 2017. Available from: https://www.canada.ca/en/health-canada/services/environmental-workplace-health/reports-publications/environmental-contaminants/national-strategic-framework-children-environmental-health-health-canada-2010.html.

[7] MIREC. MIREC Research Platform List of Publications. Available from: https://www.mirec-canada.ca/en/publications.

[8] Subbarao PAS, Becker AB, Befus AD, Brauer M, Brook JR, Denburg JA, et al. The Canadian Healthy Infant Longitudinal Development (CHILD) Study: examining developmental origins of allergy and asthma. Thorax. 2015;70:998–1000. 10.1136/thoraxjnl-2015-207246 26069286

[9] Letourneau N AF, Bell RC, Deane AJ, Dewey D, Field C, Giesbrecht G, Kaplan B, Leung B, Ntanda H; APrON Study Team. The Alberta Pregnancy Outcomes and Nutrition (APrON) longitudinal study: cohort profile and key findings from the first three years. BMJ Open. 2022;12. 10.1136/bmjopen-2020-047503. PMC8823238 35131812

[10] McDonald SW LA, Benzies KM, McNeil DA, Lye SJ, Dolan SM, Pennell CE, Bocking AD, Tough SC. The All Our Babies pregnancy cohort: design, methods, and participant characteristics. BMC Pregnancy Childbirth. 2013;13. 10.1186/1471-2393-13-S1-S2. PMC3561154 23445747

[11] Fisher MMG, Lanphear B, Arbuckle TE, Braun JM, Zidek A, Vélez MP, et al. Cohort profile update: The Canadian Maternal-Infant Research on Environmental Chemicals Child Development study (MIREC-CD PLUS). Paediatr Perinat Epidemiol. 2023;37:719–32. 10.1111/ppe.13013 37921434

[12] Government of Canada. Overview of Canadian Environmental Protection Act. 2017. Available from: https://www.canada.ca/en/environment-climate-change/services/canadian-environmental-protection-act-registry/general-information/overview.html.

[13] Government of Canada. A Right to a Healthy Environment under the Canadian Environmental Protection Act, 1999 2025 Available from: https://www.canada.ca/en/environment-climate-change/services/canadian-environmental-protection-act-registry/right-to-healthy-environment.html.

[14] Government of Canada. Implementation framework for the right to a healthy environment under the Canadian Environmental Protection Act, 1999 2025. Available from: https://www.canada.ca/en/environment-climate-change/services/canadian-environmental-protection-act-registry/publications/right-healthy-environment-implementation-framework.html.

[15] Government of Canada. Strengthening Environmental Protection for a Healthier Canada Act, 2023, c. 12 2023. Available from: https://www.parl.ca/Content/Bills/441/Government/S-5/S-5_4/S-5_4.PDF.

[16] Weihe P, P G. Cohort studies of Faroese children concerning potential adverse health effects after the mothers’ exposure to marine contaminants during pregnancy. Acta Vet Scand. 2012. 10.1186/1751-0147-54-S1-S7.

[17] P3 Cohort. 2023. Available from: https://p3cohort.ca/.

[18] MoBa. Norwegian Mother, Father and Child Cohort Study (MoBa). Available from: https://www.fhi.no/moba-en.

[19] APrON. Available from: https://apronstudy.ca/.

[20] Oken E, T B, Bornkamp N, Breton CV, Fry RC, Gold DR, et al. When a birth cohort grows up: challenges and opportunities in longitudinal developmental origins of health and disease (DOHaD) research. J Dev Orig Health Dis. 2023;14:175–81. 10.1017/S2040174422000629. PMC9998333 36408681

[21] LA Wise RK, Mikkelsen EM, Stanford JB, Wesselink AK, McKinnon C, Gruschow SM, et al. Design and conduct of an internet-based preconception cohort study in North America: pregnancy study online. Paediatr Perinat Epidemiol. 2015;4:360–71. 10.1111/ppe.12201. PMC4662659 26111445

[22] Rifas-Shiman SL, I A, Switkowski KM, Young J, Fleisch AF, Perng W, et al. Cohort profile update: project Viva offspring. Int J Epidemiol. 2024. 10.1093/ije/dyae162. PMC11630542 39657066

[23] Buck Louis GM, D B, Kannan K, Chen Z, Kim S, Sundaram R. Paternal exposures to environmental chemicals and time-to-pregnancy: overview of results from the LIFE study. Andrology. 2016;4:639–47. 10.1111/andr.12171. PMC4961554 27061873

[24] Braun JMMC, Hauser R. Fathers matter: why it’s time to consider the impact of paternal environmental exposures on children’s health. Curr Epidemiol Rep. 2017;4:46–55. 10.1007/s40471-017-0098-8. PMC5571868 28848695

[25] Harlow AF ZA, Nordberg J, Hatch EE, Ransbotham S, Wise LA. A qualitative study of factors influencing male participation in fertility research. Reprod Health. 2020;17. 10.1186/s12978-020-01046-y. PMC7684935 33228762

[26] Grace B SJ, Johnson S, Stephenson J. You did not turn up… I did not realise I was invited…: understanding male attitudes towards engagement in fertility and reproductive health discussions. Hum Reprod Open. 2019;17. 10.1093/hropen/hoz014. PMC6573469 31218265

[27] Olshan AFPS, Bradley L, Buus RM, Strader LF, Jeffay SC, et al. The healthy men study: design and recruitment considerations for environmental epidemiologic studies in male reproductive health. Fert Steril. 2006;87:554–64. 10.1016/j.fertnstert.2006.07.1517. 17140573

[28] Pekarsky C SJ, Leijser LM, Slater D, Metcalfe A. Social media recruitment strategies to recruit pregnant women into a longitudinal observational cohort study: usability study. J Med Internet Res. 2022;24. 10.2196/40298. PMC9793295 36508244

[29] Kooijman MN KC, van Duijn CM, Duijts L, Franco OH, van IJzendoorn MH, de Jongste JC, Klaver CC, van der Lugt A, Mackenbach JP, Moll HA, Peeters RP, Raat H, Rings EH, Rivadeneira F, van der Schroeff MP, Steegers EA, Tiemeier H, Uitterlinden AG, Verhulst FC, Wolvius E, Felix JF, Jaddoe VW. The Generation R Study: design and cohort update 2017. Eur J Epidemiol. 2016;31:1243–64. 10.1007/s10654-016-0224-9. PMC5233749 28070760

[30] Brandlistuen RE KD, Alsaker E, Valen R, Birkeland E, Røyrvik EC, Page CM, Aamelfot M, Vangbæk S, Ask H, Havdahl A, Brantsæter AL, Rortveit G, Håberg SE, Magnus P. Cohort Profile Update: The Norwegian Mother, Father and Child Cohort (MoBa). Int J Epidemiol. 2025;54. 10.1093/ije/dyaf139. PMC12367290 40834906

[31] Koenig MR WA, Kuriyama AS, Chaiyasarikul A, Hatch EE, Wise LA. Feasibility of mail-based biospecimen collection in an online preconception cohort study. Front Reprod Health. 2023;9. 10.3389/frph.2022.1052231. PMC9869415 36699143

[32] Mertes H HJ, Boivin J, Ekstrand Ragnar M, Grace B, Moura-Ramos M, Rautakallio-Hokkanen S, Simopoulou M, Hammarberg K, International Reproductive Health Education Collaboration Irhec OBOT. Stimulating fertility awareness: the importance of getting the language right. Hum Reprod Open. 2023;2023. 10.1093/hropen/hoad009. PMC10112336 37082102

[33] Sommer GJWT, Epperson JG, Hatch EE, Wesselink AK, Rothman KJ, Fredriksen LL, et al. At-home sperm testing for epidemiologic studies: Evaluation of the Trak male fertility testing system in an internet-based preconception cohort. Paediatr Perinat Epidemiol. 2020;34:504–12. 10.1111/ppe.12612. PMC8052852 31838751

[34] Buck Louis GM SE, Sweeney AM, Wilcosky TC, Gore-Langton RE, Lynch CD, Boyd Barr D, Schrader SM, Kim S, Chen Z, Sundaram R. Designing prospective cohort studies for assessing reproductive and developmental toxicity during sensitive windows of human reproduction and development--the LIFE Study. Paediatr Perinat Epidemiol. 2011;25:413-24. 10.1111/j.1365-3016.2011.01205.x. PMC4118054 21819423

[35] Wesselink AKHE, Rothman KJ, Weuve JL, Aschengrau A, Song RJ, Wise LA. Perceived Stress and Fecundability: A Preconception Cohort Study of North American Couples. Am J Epidemiol. 2018;187:2662–71. 10.1093/aje/kwy186. PMC6887691 30137198

[36] Lambert T, Stephenson N, Skiffington J, Slater D, Leijser LM, Metcalfe A. Impact of couple vs. individual participation in pregnancy research: A comparative analysis of participant characteristics and study retention. Ann Epidemiol. 2025;111:163–7. 10.1016/j.annepidem.2025.10.013. 41077159

[37] Fazli GS PE, Crighton E, Sarwar A, Ashley-Martin J. Engaging, recruiting, and retaining pregnant people from marginalized communities in environmental health cohort studies: a scoping review. BMC Public Health. 2025;25. 10.1186/s12889-025-22033-7. PMC11887406 40050851

[38] Aiyegbusi OL, Cruz Rivera S, Roydhouse J, et al. Recommendations to address respondent burden associated with patient-reported outcome assessment. Nat Med. 2024;30:650–9. 10.1038/s41591-024-02827-9. 38424214

[39] Aiyegbusi OLRJ, Rivera SC, Kamudoni P, Schache P, Wilson R, Stephens R, et al. Key considerations to reduce or address respondent burden in patient-reported outcome (PRO) data collection. Nat Commun. 2022;13:6026. 10.1038/s41467-022-33826-4. PMC9556436 36224187

[40] Dirks A, Botto E, Smith Z, et al. Trends in the burden for patients participating in industry-funded clinical trials. Ther Innov Regul Sci. 2025;59:893–900. 10.1007/s43441-025-00805-y. 40484896

[41] Getz K, Sethuraman V, Rine J, et al. Assessing patient participation burden based on protocol design characteristics. Ther Innov Regul Sci. 2020;54:598–604. 10.1177/2168479019867284. 33301141

[42] PEHE. Reimagining Research. 2022. Available from: https://www.pehe-esep.ca/reimagining-research.

[43] Israel B, Lichtenstein R, Lantz P, McGranaghan R, Allen AM, Guzman R, et al. The Detroit Community-Academic Urban Research Center: Development, Implementation, and Evaluation. J Public Health Management Practice. 2001;7:1-19. 10.1097/00124784-200107050-00003. 11680026

[44] Israel B, Schulz AJ, Parker EA, Becker, AB. Review of community-based research: Assessing partnership approaches to improve public health. Annu Rev Public Health. 1998;19. 10.1146/annurev.publhealth.19.1.173. 9611617

[45] Phelan KJKJ, Xu Y, Liddy S, Hornung R, Lanphear BP. A randomized controlled trial of home injury hazard reduction: The HOME injury study. Arch Pediatr Adolesc Med. 2011;165:339–45. 10.1001/archpediatrics.2011.29. PMC3693223 21464382

[46] Comachio JPA, Bamgboje-Ayodele A, et al. Identifying and counteracting fraudulent responses in online recruitment for health research: a scoping review. BMJ Evid Based Med. 2025;30:173–82. 10.1136/bmjebm-2024-113170. PMC12171401 39715631

[47] NIH. Environmental influences on Child Health Outcomes (ECHO) Program [Available from: https://www.nih.gov/research-training/medical-research-initiatives/environmental-influences-child-health-outcomes-echo-program. 10.1038/s41366-019-0470-5 PMC7060502 31649277

[48] Green JM BF, Burton P, Beauchemin J, Huentelman MJ, Deoni SCL, Lewis CR; program collaborators for Environmental Influences on Child Health Outcomes. At-home dried blood spot (DBS) collection to increase population heterogeneity representation in pediatric research: An ECHO study. Front Pediatr. 2023;11. 10.3389/fped.2023.1059107. PMC10020170 36937973

[49] Braun JM KG, Chen A, Dietrich KN, Liddy-Hicks S, Morgan S, Xu Y, Yolton K, Lanphear BP. Cohort Profile: The Health Outcomes and Measures of the Environment (HOME) study. Int J Epidemiol. 2017;46. 10.1093/ije/dyw006. PMC5837495 27006352

[50] Wise LAWT, Willis SK, Wesselink AK, Rothman KJ, Hatch EE. Effect of a home pregnancy test intervention on cohort retention and pregnancy detection: A randomized trial. Am J Epidemiol. 2020;189:773–8. 10.1093/aje/kwaa027. PMC7407601 32128561

[51] Wise LAWT, Stanford JB, Wesselink AK, Ncube CN, Rothman KJ, Murray EJ. A randomized trial of web-based fertility-tracking software and fecundability. Fertil Steril. 2023;119:1045–56. 10.1016/j.fertnstert.2023.02.005. PMC10225320 36774978

[52] O’Brien KMLK, Keil AP. The case for case-cohort: An applied epidemiologist’s guide to reframing case-cohort studies to improve usability and flexibility. Epidemiology. 2022;22:354–61. 10.1097/EDE.0000000000001469. PMC9172927 35383643

[53] Haviland MJNY, Fox MP, Savitz DA, Hatch EE, Rothman KJ, Hacker MR, et al. Psychotropic medication use during pregnancy and gestational age at delivery. Ann Epidemiol. 2021;53:34–41. 10.1016/j.annepidem.2020.08.010. PMC7736493 32835770

[54] Wise LAWT, Wesselink AK, Willis SK, Chaiyasarikul A, Levinson JS, Rothman KJ, et al. . Accuracy of self-reported birth outcomes relative to birth certificate data in an Internet-based prospective cohort study. Paediatr Perinat Epidemiol. 2021;35:590–5. 10.1111/ppe.12769. PMC8380669 33956369

[55] Grace B, Nieroda M, Egbunike J, Usman N. Digital health technologies to transform women’s health innovation and inclusive research. BMJ. 2025. 10.1136/bmj-2025-085682. PMC12509992 41073079

[56] Haines DA AT, Lye E, Legrand M, Fisher M, Langlois R, Fraser W. Reporting results of human biomonitoring of environmental chemicals to study participants: a comparison of approaches followed in two Canadian studies. J Epidemiol Community Health. 2011;65. 10.1136/jech.2008.085597. 20628082

[57] Katrina Smith Korfmacher 1 JGB. Moving Forward with Reporting Back Individual Environmental Health Research Results. Environ Health Perspect. 2023;131. 10.1289/EHP12463. PMC10720702 38095662

[58] NASEM. Returning Individual Research Results to Participants: Guidance for a New Research Paradigm. Washington, DC; 2018. 30001048

[59] Silent Spring Institute. Digital Exposure Report-Back Interface (DERBI) 2025. Available from: https://silentspring.org/project/digital-exposure-report-back-interface-derbi.

[60] Mash C, McAllister KA, Wonnum S, Vargas AJ, Dowling G, Arteaga SS, et al. Principles and practices of returning individual research results to participants in large studies of pregnancy and childhood. Am J Epidemiol. 2025;194:830. 10.1093/aje/kwae228. PMC12491858 39030726

[61] Samuels SWRK, Rom WN, Frank A. Ethical thinking in occupational and environmental medicine: commentaries from the Selikoff Fund for Occupational and Environmental Cancer Research. Am J Ind Med. 2022;65:286–320. 10.1002/ajim.23328. PMC9302668 35156722

[62] Savitz DA, Poole C, Olshan AF. Epidemiologic measures of the course and outcome of pregnancy. Epidemiol Rev. 2002;24:91–101. 10.1093/epirev/mxf006. 12762085

[63] Messerlian C, Williams PL, Ford JB, Chavarro JE, Mínguez-Alarcón L, Dadd R, et al. The Environment and Reproductive Health (EARTH) Study: A Prospective Preconception Cohort. Hum Reprod Open. 2018;2018:hoy001. 10.1093/hropen/hoy001. PMC5990043 29888739

[64] Dennis CL, Marini F, Prioreschi A, Dol J, Birken C, Bell RC. The Canadian Healthy Life Trajectories Initiative (HeLTI) Trial: a study protocol for monitoring fidelity of a preconception-lifestyle behaviour intervention. Trials. 2023;24:262. 10.1186/s13063-023-07271-7. PMC10079485 37024918

[65] Vernet C, Philippat C, Calafat AM, Ye X, Lyon-Caen S, Siroux V, et al. Within-day, between-day, and between-week variability of urinary concentrations of phenol biomarkers in pregnant women. Environ Health Perspect. 2018;126:037005. 10.1289/EHP1994. PMC6071804 29553460

[66] Quenby SGI, Dhillon-Smith RK, Podesek M, Stephenson MD, Fisher J, Brosens JJ, et al. Miscarriage matters: the epidemiological, physical, psychological, and economic costs of early pregnancy loss. Lancet. 2021;397:1658–67. 10.1016/S0140-673(21)00682-6. 33915094

[67] Kolte AMWD, Lidegaard Ø, Brunak S, Nielsen HS. Chance of live birth: a nationwide, registry-based cohort study. Hum Reprod. 2021;36:1065–73. 10.1093/humrep/deaa326. 33394013

[68] Ashley-Martin J, Hammond J, Velez MP. Assessing preconception exposure to environmental chemicals and fecundity: Strategies, challenges, and research priorities. Reprod Toxicol. 2024;125:108578. 10.1016/j.reprotox.2024.108578. 38522558

[69] Bradford Hill A. The environment and disease: association or causation? Proc R Soc Med. 1965;58:295–300. 10.1177/003591576505800503. PMC1898525 14283879

[70] Wilkinson MD, Dumontier M, Aalbersberg IJ, Appleton G, Axton M, Baak A, Blomberg N, Boiten JW, da Silva Santos LB, Bourne PE, Bouwman J, Brookes AJ, Clark T, Crosas M, Dillo I, Dumon O, Edmunds S, Evelo CT, Finkers R, Gonzalez-Beltran A, Gray AJ, Groth P, Goble C, Grethe JS, Heringa J, 't Hoen PA, Hooft R, Kuhn T, Kok R, Kok J, Lusher SJ, Martone ME, Mons A, Packer AL, Persson B, Rocca-Serra P, Roos M, van Schaik R, Sansone SA, Schultes E, Sengstag T, Slater T, Strawn G, Swertz MA, Thompson M, van der Lei J, van Mulligen E, Velterop J, Waagmeester A, Wittenburg P, Wolstencroft K, Zhao J, Mons B. The FAIR Guiding Principles for scientific data management and stewardship. Sci Data. 2016;15:160018. 10.1038/sdata.2016.18. PMC4792175 26978244

[71] Puvvula J, Hwang WT, McCandless L, Xie C, Braun JM, Vuong AM, et al. Gestational exposure to environmental chemical mixtures and cognitive abilities in children: a pooled analysis of two North American birth cohorts. Environ Int. 2025;196:109298. 10.1016/j.envint.2025.109298. PMC11896093 39893913

[72] Li NAT, Muckle G, Lanphear BP, Boivin M, Chen A, Dodds L, et al. Associations of cord blood leptin and adiponectin with children’s cognitive abilities. Psychoneuroendocrinology. 2019;99:257–64. 10.1016/j.psyneuen.2018.10.021. PMC6239208 30390444

[73] Raina PWC, Kirkland S, Griffith LE, Balion C, Cossette B, Dionne I, et al. Cohort profile: The Canadian Longitudinal Study on Aging (CLSA). Int J Epidemiol. 2019;48:1752–3. 10.1093/ije/dyz173. PMC6929533 31633757

[74] Dummer TJ, Awadalla P, Boileau C, Craig C, Fortier I, Goel V, et al. The Canadian Partnership for Tomorrow Project: a pan-Canadian platform for research on chronic disease prevention. CMAJ. 2018;190:E710-7. 10.1503/cmaj.170292. PMC5995593 29891475

[75] Thompson LCHK, Vogel AL, Park CH, Gillman MW. Conceptual models for implementing solution-oriented team science in large research consortia. J Clin Transl Sci. 2021;5:e139. 10.1017/cts.2021.802. PMC8327547 34367683

[76] Röhnisch HE, Kyrø C, Olsen A, Thysell E, Hallmans G, Moazzami AA. Identification of metabolites associated with prostate cancer risk: a nested case-control study with long follow-up in the Northern Sweden Health and Disease Study. BMC Med. 2020;18:187. 10.1186/s12916-020-01655-1. PMC7376662 32698845

[77] Strand KM, Odland ML, Iversen AC, Nordbø SA, Vik T, Austgulen R. Cytomegalovirus antibody status at 17–18 weeks of gestation and pre-eclampsia: a case-control study of pregnant women in Norway. BJOG. 2012;119:1316–23. 10.1111/j.1471-0528.2012.03420.x. 22804776

[78] Wesselink AK, Weuve J, Fruh V, Bethea TN, Claus Henn B, Harmon QE, et al. Urinary concentrations of phenols, parabens, and triclocarban in relation to uterine leiomyomata incidence and growth. Fertil Steril. 2021;116:1590–600. 10.1016/j.fertnstert.2021.07.003. PMC8627427 34366109

[79] Wacholder S. Practical considerations in choosing between the case-cohort and nested case-control designs. Epidemiology. 1991;2:155–8. 10.1097/00001648-199103000-00013. 1932316

[80] McGee G, Perkins NJ, Mumford SL, Kioumourtzoglou MA, Weisskopf MG, Schildcrout JS, et al. Methodological issues in population-based studies of multigenerational associations. Am J Epidemiol. 2020;189:1600–9. 10.1093/aje/kwaa125. PMC7822644 32608483

